# Blood Pressure in Tuberculosis

**Published:** 1919-03

**Authors:** W. O. Wilkes

**Affiliations:** Waco, Texas


### Blood Pressure in Tuberculosis.

By W. O. WILKES, M. D., WACO, TEXAS.

It is a well known fact that blood pressure is much below normal in tuberculous subjects, even in the very earliest stages. It may be possible that low blood pressure from other causes may be an exciting cause in bringing about an activity in the old tuberculous foci which most individuals carry concealed about their person, for "If there is a continuous low systemic arterial blood pressure the circulation is always more or less imperfect, and the physical ability of the individual is below par.* * * A persistently low blood pressure may be of serious import. * * * * If possible, the cause should be determined and the condition improved." (J. A. M. A., July 1, 1916, p. 34). Fishberg says, (p. 222), that when a low blood pressure is found "in an adult without any other assignable cause tuberculosis is to be suspected." I am inclined to think that while this statement is measurably true it might be better to change the word "suspected" to "expected," and say that when low blood pressure is found in an adult without other assignable cause tuberculosis is to be expected, unless the condition can be corrected. However, either word may be correct—or both—suspected and expected.

John Ritter "has found, (Trans. Nat. Assn. Study and Prev. Tuber. 1911, VII, 297.), hypotension in case of phthisis before physical signs, and even before elevation of temperature, were definitely demonstrable." Whether it is a cause or an effect, or both as I believe it to be, the fact remains that all tuberculous subjects have low blood pressure, unless at the same time they have cardiovascular-renal disease. Just how low this may be expected to be is stated differently by different observers; but it is probable that these differences may be largely due to different methods of taking blood pressure readings. There are so many different factors entering into the rise and fall of blood pressure findings in normal as well as sick persons that no set of observations has any value unless the observer's methods are set forth; such as the position of the patient, whether sitting, standing or reclining; the kind of instrument and arm-band used; whether the right or left arm; whether before or after exercise, meals or medicines; the patient's psychic state, and so forth. All these things will effect the blood pressure reading, also the individual peculiarities of the observer must be taken into consideration, as to whether he uses the fingers at the wrist, or a stethoscope on the arm, or an arm bracelet; and whether he makes his systolic reading with the first pulse sound heard or later when the heart sound becomes clear, regular and rythmic.

In the Journal of the American Medical Association for August 17, 1918, a paper was published by Brooks and Bleile, of Columbus, advocating the systematizing of blood pressure readings made by the auscultatory method. They divide the blood pressure scale into eight phases of sound: 1. Silence, when the cuff has been distended until all sound is cut off. 2. Murmurs when air has been allowed to slowly escape until indistinct sounds are heard. This phase extends over a narrow limit of 5 to 10 mm. 3. Arythmic sounds, or Desultory Snapping sounds, or "Thumps." 4. Regular Rhythmic Snapping Sounds. 5. Friction Sounds, or "Thump-sh-sh," the sounds gradually growing louder. 6. Regular Snapping Sounds gradually growing less in intensity. 7. The phase of murmurs again. 8. Silence. They wish to establish the systolic reading at the beginning of the regular, rhythmic snapping sounds, or the beginning of the 4th phase, and the diastolic at the point where the regular, rhythmic snapping sounds cease, or at the close of the 6th phase. Obviously, this would make the pulse pressure much narrower in range, as the systolic reading will not be so high as in the ordinary method of taking it, and the diastolic possibily not so low. If some such method as this could be universally adopted it would give much greater value to blood pressure statistics.

Fishberg says (p. 311), "a registration less than 70 mm. of mercury is quite common" in incipient phthisis, but I have not found it so. I have never seen a systolic blood pressure below 80 mm. in any adult individual, but have found many tuberculous patients with a systolic pressure between 80 and 90. I take the reading by ear with a Bowles arm bracelet, using a five-inch arm band on the left arm with the patient seated in a comfortable position. Then I call the systolic pressure at the point where the first pulse is heard on letting the air escape slowly from the distended arm band, and the diastolic the last distinct sound heard. This method gives a wide pulse pressure and a higher systolic than the finger on the wrist. The ideal normal ratio between the pulse pressure, the diastolic and the systolic should be approximately 1-2-3. If Fishberg used the old method of feeling for the pulse at the wrist he may have found a systolic of less than 70 in many incipient cases, if the ends of his fingers are very insensitive.

Pottenger says (p. 235): "Blood pressure is lowered early in the disease, although wide departures from the normal are rarely seen except in advanced cases."

So, there you are. Fishberg was published in 1916, and Pottenger in 1917, so both should be about up-to-date, yet these statements are directly opposed. Fishberg also says lowered blood pressure "is evidently due to the toxic effects of the metabolic processes of the tubercle bacilli." Pottenger says: "It is generally stated that toxins reduce the blood pressure. This is untrue of toxins per se unless the toxaemia is very severe. Toxins act upon the vasomotor center through the sympathetic system and produce vasoconstriction"—otherwise, hypertension.

I am nobody in this controversy, but I believe the hypotension is caused by general physical weakness, plus possibly some peculiar quality of the tubercular toxins; and that hypotension may be one cause for activity, and is always a sequel of tuberculosis.

Pottenger in his book claims to have made a study of blood pressure in 162 tuberculous patients and 20 non-tuberculous individuals. His 20 non-tuberculous individuals showed an average systolic pressure of 120 mm., and diastolic 108. They, to my mind, are twenty curious individuals with only 12 mm. difference between the systolic and diastolic pressures. This statement renders worthless his studies of the 162 tuberculous patients. He should have described his methods, and then we might understand how the so-called pulse pressure is only 12 mm., showing a ratio between it and the diastolic and the systolic of 1-9-12, instead of 1-2-3, which is the ideal normal when taken by the auscultatory method. He also makes the remark that "82 mm. of mercury is the point of syncope," so that Fishberg's many patients with a pressure below 70 mm. must have been in a deep state of syncope. I cannot find where Pottenger got his "point of syncope;" and I cannot account for Fishberg's many incipient cases below 70 mm. systolic.

About two years ago I began taking blood pressure records of all tuberculous patients who came to my office, by the auscultatory method, using a Tycos instrument and a Bowles arm bracelet. The general systolic average of 319 patients examined in that period was 107.2 mm. of mercury, and the diastolic 70.3 mm., giving a pulse pressure of 36.9. This is very close to the ideal normal ratio of 1-2-3, but both the systolic and diastolic readings are, of course, much below the normal for normal individuals. Burckhardt, of Basel, gives his findings for the systolic as 107.6 in incipient cases, 104.6 in second stage and 100.3 in third stage cases. This corresponds closely to my findings. I have made no effort to divide my cases into first, second and third stages for this study (the personal equation might come in here strongly), but they were all cases who came to my office for examination, and I estimate that they were about 50 to 60 per cent incipient, 30 to 40 per cent, second stage and only about 10 per cent third stage cases. Few third stage cases come to my office. My third stage cases, I am sure, will average below Burckhardt's findings of 100.3 mm. These patients ranged in age from 16 to 62. If cases known to be complicated with cardiovascular-renal disease had been excluded from the 319, the average of 107.2 would have been somewhat reduced, possibly to 105, but I have found that patients with cardiovascular-renal disease and tuberculosis coexistent do not have nearly so high a blood pressure as do the ordinary cases of cardiovascular-nenal disease without tuberculosis, my few cases showing a systolic pressure of less than 150. Furthermore, I do not believe that cardiovascular-renal diseases often occur in tuberculous subjects, although heart degeneration does often occur.

I am naturally partial to the method I have been using for the last two years because I am now accustomed to it, but chiefly because of the expected 1-2-3 ratio, which, if materially disturbed, causes me to search for the reason in the cardiovascular-renal system, if it cannot be accounted for by psychic or other influences of a transitory nature.

I do not believe that low blood pressure has any particular prognostic significance in tuberculosis, because some of the most favorably progressing incipient cases I have had under my care have shown a very low blood pressure in the beginning. Yet, we are always most hopeful of those cases whose blood pressure is nearest the normal. When a blood pressure is low at first and then gradually rises it is looked upon as a good indication; and when a blood pressure steadily falls under treatment it certainly gives a very grave prognosis.

Finally, I wish to say, as has been so often said before, that there seems to be much yet to be learned about blood pressure and its relations to health and disease; but we do know that a very low systolic pressure without assignable cause points conclusively to tuberculosis, present or to come.

